# Fabrication and Evaluation of Basil Essential Oil-Loaded Halloysite Nanotubes in Chitosan Nanocomposite Film and Its Application in Food Packaging

**DOI:** 10.3390/antibiotics11121820

**Published:** 2022-12-15

**Authors:** Narayan Chaudhary, Gourav Mishra, Tushar Yadav, Nishant Srivastava, Vimal K. Maurya, Shailendra K. Saxena

**Affiliations:** 1 Nanotoxicity & Drosophila Research Laboratory, Department of Biotechnology, Meerut Institute of Engineering and Technology, Meerut 250005, India; 2 Department of Zoology, Jawaharlal Nehru Smriti Government Postgraduate College, Shujalpur 465333, India; 3 Centre for Advanced Research (C.F.A.R.), Faculty of Medicine, King George’s Medical University (KGMU), Lucknow 226003, India

**Keywords:** nanotubes, essential oil, natural polymer, antioxidant, active packaging

## Abstract

Increasing health concerns regarding the use of plasticware have led to the development of ecofriendly biodegradable packaging film from natural polymer and food additives. In the present study, basil essential oil (BEO) loaded halloysite nanotubes (HNTs) composite films were synthesized using a solution casting method. The effects of BEO and nanotube concentration on the mechanical, physical, structural, barrier, and antioxidant properties of films were evaluated. Scanning electron microscopy (SEM), X-ray diffraction (XRD) and Fourier transform infrared (FTIR) demonstrated well-dispersed HNTs and BEO in tailored composite films. The addition of BEO in Chitosan (Ch) film caused darkening of the film color; furthermore, the incorporation of HNTs in varied concentrations increased opaqueness in Ch/BEO film. The Ch/BEO film, upon adding HNTs 5–30 wt%, exhibited a corresponding increase in the film thickness (0.108–0.135 mm) when compared with the Ch/BEO film alone (0.081 mm). The BEO-loaded HNTs composite films displayed reduced moisture content and characteristic barrier and UV properties. The Ch/BEO film with 15 wt% HNTs was found to have enhanced antioxidant activity. The Ch/BEO/HNTs composite also managed to prevent broccoli florets from losing weight and firmness during storage. The enhanced barrier and antioxidant qualities of the nanocomposite film suggest its potential application in the food processing and packaging sector. This is the first ever report on the fabrication of nanocomposite film using BEO and HNTs for food packaging. The low production cost and ecofriendly approach make the film acceptable for further research and commercialization thereafter.

## 1. Introduction

Food packaging plays a significant role in modern society and is considered an integral aspect of the food processing and packaging sector. Packaging prevents damage during transit, maintains food quality, and influences the safety, preservation, and extension of the shelf life of food [1,2]. Packaging protects foods from environmental influences such as light, humidity, temperature, bacteria, dust, and compressive pressures [3]. While plastic packaging is now the most common type of food packaging, environmental concerns and consumer demand for an ecofriendly alternative have grown over the past few decades. There has been great interest in biodegradable active packaging because of its outstanding biodegradability, biocompatibility, and prospective uses [4,5].

Among natural biopolymers, chitosan (Ch) stands out as the most promising candidate for active packaging materials and is “generally recognized as safe” (GRAS). Ch is a polysaccharide composed of β-1, 4-linked glucosamine, and N-acetylglucosamine, obtained from the deacetylation of chitin [6]. After cellulose, it is the polysaccharide with the highest prevalence among natural macromolecules. It can be derived from natural sources such as crustacean shells, shrimp, and crabs [7]. Because of its nontoxicity, biodegradability, biocompatibility, and antibacterial and antioxidant activity, Ch may prove to be an appropriate material for food packaging. Furthermore, the ability to form films makes it a suitable biopolymer for food packaging. Due to the positively charged amino group, Ch has natural antibacterial properties [8]. Because of its hydrophilic nature, Ch imparts a weak moisture barrier [9]. To improve the characteristics of Ch films, they must be mixed with nanomaterials such as nanoclay and infused with essential oils [10,11]. The addition of essential oils to biofilms improves food safety by extending antibacterial and antioxidant characteristics. Essential oils are hydrophobic compounds used to improve the water resistance qualities of hydrophilic hydrocolloid films [12]. The concentration of essential oils is high in liquids derived from plants or spices. They have volatile aromatic molecules and are organic. Essential oils are nontoxic to the environment [13], are composed of substances, especially phenolic compounds that minimize the phenomena of lipid oxidation, and have antibacterial and antioxidant action or reduce the presence of microbes [14]. As a result, they can help to decrease or even eliminate the need for synthetic additives. Consumers are becoming more aware of the deleterious effects of synthetic additives on human health, and they are beginning to turn against items that contain additives that are not natural [15].

Basil (*Ocimum basilicum* L.) essential oil (BEO) has been used as an antibacterial and antioxidant agent in food or food packaging, and it is generally recognized as safe (GRAS) by the Food and Drug Administration (FDA) [16]. Basil is an aromatic, branching annual herb with antioxidant properties that has been used as a culinary herb and in traditional medicine [17]. According to studies, basil has anti-inflammatory, analgesic, immunomodulatory, hepatoprotective, and antibacterial properties. In many products, BEO is used as a food additive to prevent oxidation, as an antibacterial agent, or as a flavor and aroma modifier [18].

Halloysite nanotubes (HNTs) are naturally aluminosilicate clay nanotubes (Al_2_Si_2_O_5_(OH)_4_*n*H_2_O) with a similar chemical composition to kaolinite. HNTs exhibit different chemical characteristics between the internal (Al-OH) and external surface (Si-O-Si). Thanks to their unique hollow morphology and large cavities, HNTs can be employed as an ideal natural nanocarrier. They are known to enhance the properties of the polymer matrix composites when effectively dispersed in the polymer matrix; additionally, the US Food and Drug Administration has approved them as a food packaging material that is safe (GRAS) [19,20,21]. Due to their nonhazardous nature, excellent biocompatibility, and easy availability, these nanotubes offer unique qualities that may be widely exploited for biological applications in a diverse variety of areas [22]. Furthermore, HNTs have also been investigated as a nanomaterial for the development of nanocomposites, and used nanofiller as a carrier for active ingredients [23,24,25,26,27]. Because of their high aspect ratio structure, HNTs act as stabilizers to encapsulate the essential oil in a mixed matrix polymer solution [28]. When HNTs are introduced to a polymer matrix, they create tortuous routes for molecules of gas and vapors to pass through, which improves the polymer boundary properties [29,30,31]. The high volatility of essential oils is a drawback of utilizing them in food packaging; HNTs act to capture the essential oils for prolonged release [32].The objective of this study was to develop BEO-loaded HNTs nanocomposite films using a solution casting method, and evaluate their mechanical, physical, structural, barrier, and antioxidant properties. To check the applicability of fabricated Ch/BEO/HNTs nanocomposite film in food packaging, the quality attributes of broccoli florets were evaluated during their storage in nanocomposite film. This study presents unique experimental data on the production of ecofriendly food packaging material.

## 2. Results and Discussion

### 2.1. Physical Properties of Film

#### 2.1.1. Thickness

**Figure 1:**
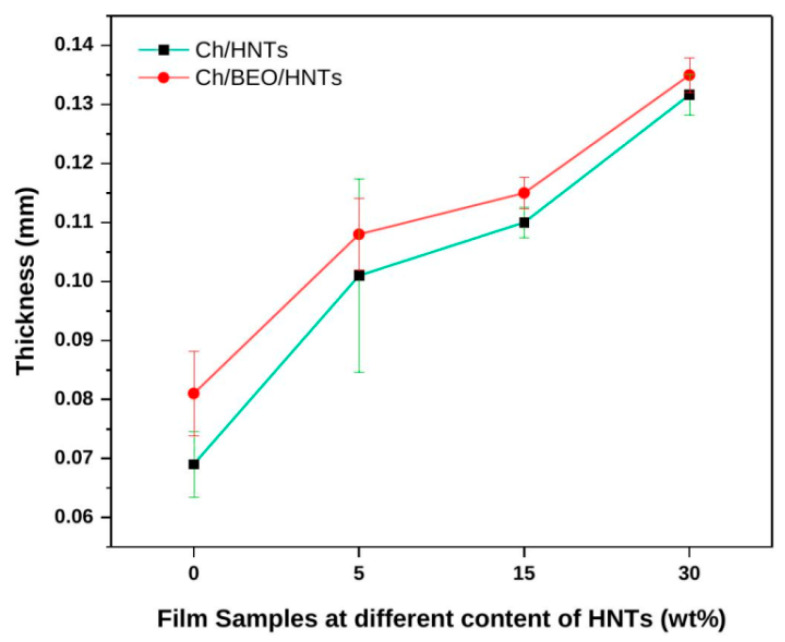
Thickness of Ch films formulated with HNTs and BEO.

Our results suggest that as the quantity of HNTs in the film increases, the Ch/HNTs film’s thickness increases marginally (Figure 1). Without BEO, the film thickness ranged from 0.069 to 0.13 mm. The inclusion of BEO in nanocomposite films raised the film thickness significantly (*p* < 0.05). This outcome is consistent with previous research studies [33,34]. The Ch/BEO film with HNTs 30 wt% was found to be the thickest one (0.135 mm). When HNTs (5–30 wt%) were added, the thickness of the film (0.108–0.135 mm) drastically increased in comparison with the Ch/BEO film’s thickness. In the current investigation, BEO was dispersed in the Ch solution used to create the film using ultrasonic treatment. After the ultrasonic treatment, the BEO droplets were initially tiny and equally distributed in the Ch dispersion used to create films, but due to its hydrophobicity, the BEO might aggregate into larger droplets during the drying process, enhancing the film’s thickness [5].

**Figure 2:**
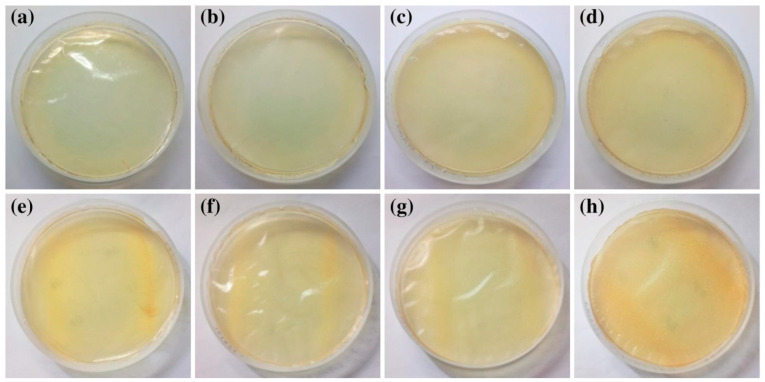
**Representative images of nanocomposite films.** (**a**) Ch film; (**b**) Ch/HNTs—5%; (**c**) Ch/HNTs—15%; (**d**) Ch/HNTs—30%; (**e**) Ch/BEO; (**f**) Ch/BEO/HNTs—5%; (**g**) Ch/BEO/HNTs—15%; (**h**) Ch/BEO/HNTs—30%.

Each nanocomposite film was visually perfect and devoid of any obvious flaws, as seen in Figure 2. The bare Ch film had a cream-colored tinge and was translucent. However, with the incorporation of different HNT concentrations into the Ch film, the film’s color transitioned from a cream tint to light yellow, and the transparency of the film also varied. The color of the film changes from light yellow to yellow when BEO is incorporated into the Chfilm, and the film becomes more opaque as HNTs of varying concentrations are incorporated into the Ch/BEO film.

#### 2.1.2. Moisture Content

**Figure 3:**
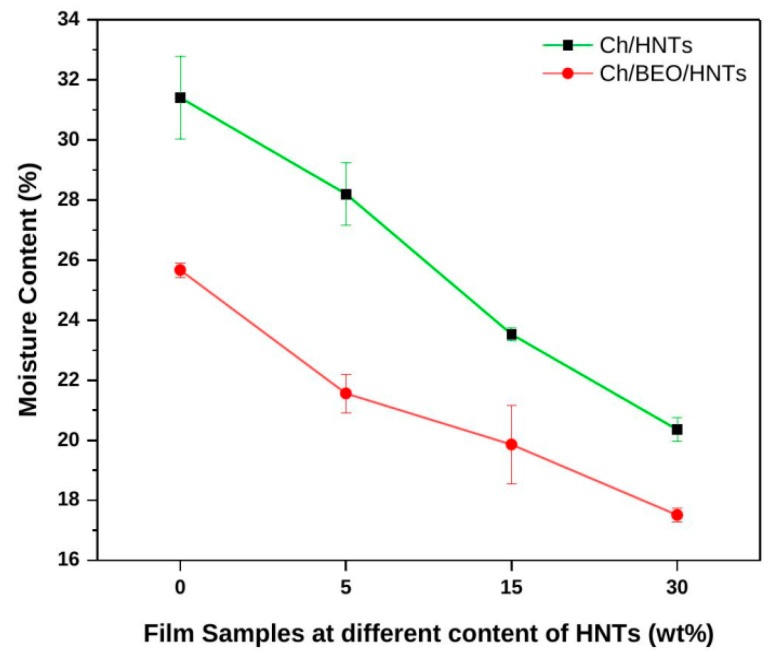
Moisture content of Ch films formulated with HNTs and BEO.

The moisture content of nanocomposite films was significantly decreased (*p* < 0.05) by the addition of BEO (Figure 3). This could be the result of covalent bonds between BEO and the functional groups of the polymer chains, which result in lower levels of free hydroxyl and amino groups. As a result, the water molecules’ hydrogen-bonded interactions with the polymer matrix are reduced. Furthermore, adding HNTs to the Ch film changed the moisture content of the film. Particularly while increasing HNT concentration, the moisture content of Ch/HNTs films without BEO decreased considerably (*p* < 0.05). While water absorption is reduced by the particles of HNTs in the film matrix, neat Ch films have a greater water attraction than Ch/HNTs nanocomposite films. A similar result is consistent with previous research studies [6,11].

#### 2.1.3. Water Absorption

**Figure 4:**
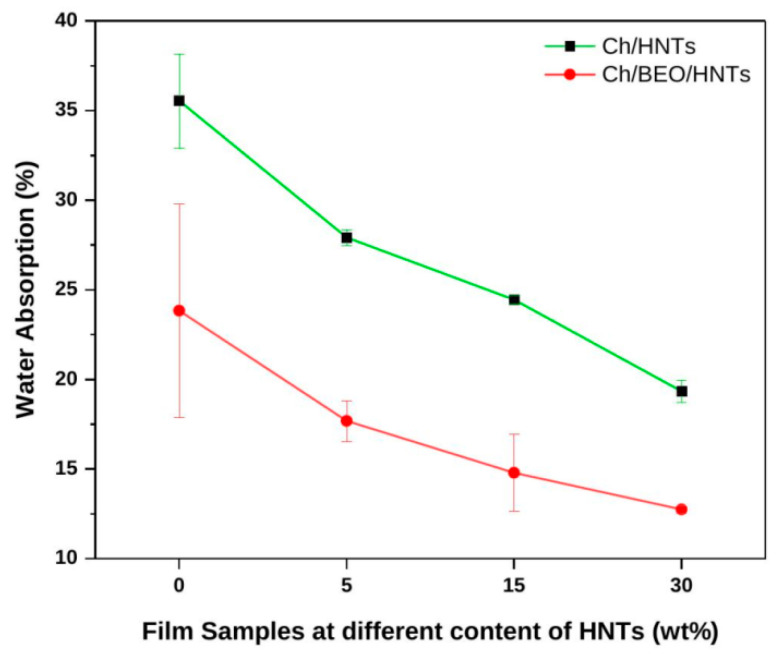
Water absorption of Ch films formulated with HNTs and BEO.

The water absorption properties of nanocomposite films are shown in Figure 4. The bare Ch film quickly undergoes hydrogen bonding with water, and swells by up to 35.55%. When it comes to Ch/HNTs, as the quantity of HNTs increases (5–30 wt%) the water absorption of the film decreases (27.92–19.33%), due to the strong interaction of HNTs with Ch functional groups, which minimizes the hydrogen bonds involved in the reaction. Water absorption in the Ch/BEO/HNTs nanocomposite films decreased (23.83–12.74%) as the HNT concentration increased. Furthermore, compared with films without BEO, film samples with BEO demonstrate lower water absorption. This is because BEO improves the hydrophobicity of the film’s surface and inhibits moisture uptake, resulting in lower absorption of water [5]. The results were found to be significant at *p* < 0.05.

#### 2.1.4. Solubility

**Figure 5:**
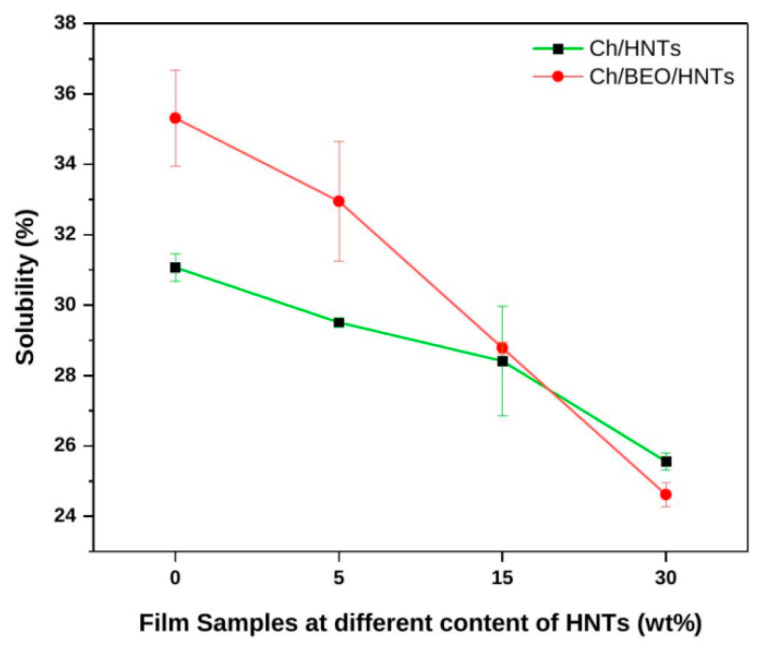
Solubility of Ch films formulated with HNTs and BEO.

The solubility of nanocomposite films is shown in Figure 5. When compared with other films, the bare Ch film demonstrates a greater affinity (31.06%) and a higher rate of solubilization in water, because Ch absorbs water quickly due to being more hydrophilic. Water solubility significantly decreases (*p* < 0.05) from 29.50 to 25.55% as HNT content (5–30%) increases in the Ch film, as HNTs interact more efficiently with the polymer matrix and the number of hydrophilic groups in the polymer matrix is lowered. On addition of BEO to a bare nanocomposite film, the water solubility of the film increases to 35.31%. Incorporation of BEO into the Ch/HNTs film decreases (32.95–24.62%) the water solubility of the film. This is because the film contains a hydrophobic component that prevents water from being absorbed. This may be due to BEO molecules migrating from the Ch film matrix into the water [5].

### 2.2. Scanning Electron Microscope (SEM)

**Figure 6:**
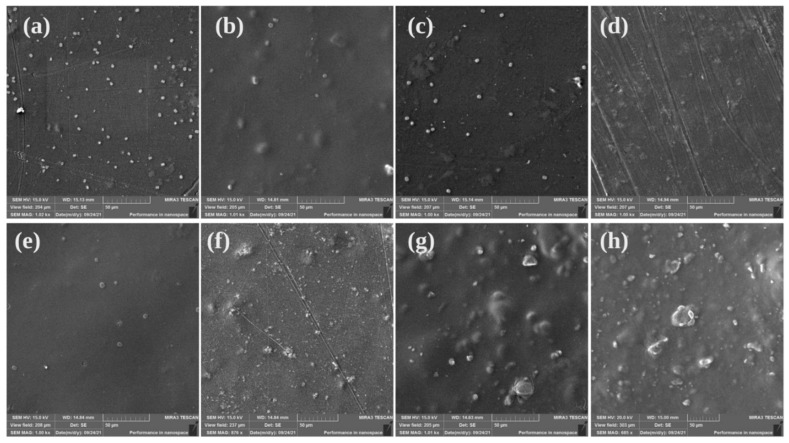
**Scanning Electron Microscopy (SEM) images of the films.** (**a**) Ch film; (**b**) Ch/HNTs—5%; (**c**) Ch/HNTs—15%; (**d**) Ch/HNTs—30%; (**e**) Ch/BEO; (**f**) Ch/BEO/HNTs—5%; (**g**) Ch/BEO/HNTs—15%; (**h**) Ch/BEO/HNTs—30%.

SEM analysis was performed to examine the surface morphologies of films made of Ch, Ch/BEO, and Ch/BEO/HNTs (with varying amounts of HNTs). Figure 6 illustrates the SEM images. The bare Ch film was uniform and compact, with no phase separation, and a smooth surface free of cracks and pores. When BEO was added to the films, the structure became denser, showing the equal distribution of oil droplets into the polymer matrix. The surface was practically homogenous and smooth, with only a few streaks or irregular lines visible. When the dispersed BEO compounds agglomerated, the inclusion of BEO caused micropores in the film matrix, as well as coalescence and flocculation of droplets of BEO which occurred when the films dried, as described earlier [35,36]. The BEO’s stability might be increased due to the concentration and size of HNTs that may have caused the reduction of pores on the film’s surface (Figure S1) [5]. The presence of HNT particles that were scattered throughout the Ch matrix appears to prevent BEO molecules from clumping together.

### 2.3. Contact Angle

**Figure 7:**
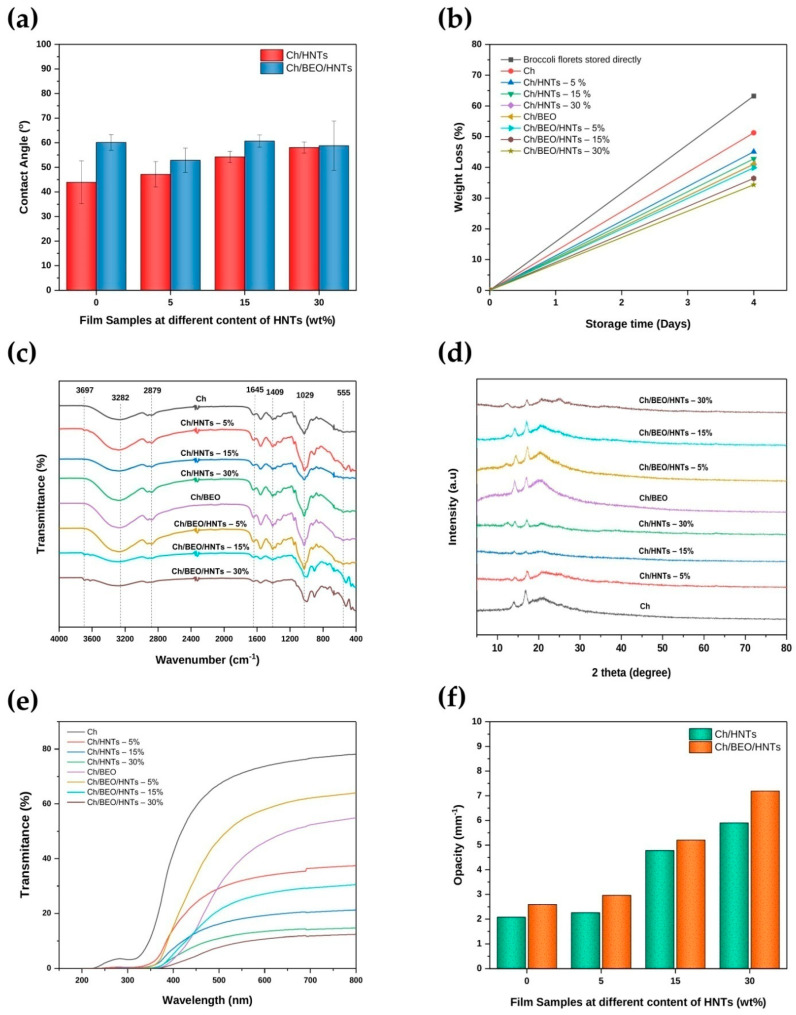
**Figures representing the** (**a**) contact angle of Ch films formulated with HNTs and BEO; (**b**) weight loss during 4 days storage; (**c**) ATR–FTIR spectrum of Ch films formulated with HNTs and BEO; (**d**) XRD pattern analysis of Ch films formulated with HNTs and BEO; (**e**) UV transmittance of Ch films formulated with HNTs and BEO; (**f**) opacity of Ch films formulated with HNTs and BEO.

Figure 7a depicts the contact angle of the film. The contact angle of a liquid droplet in the air on the surface of each film was measured to investigate the impact of BEO and HNTs on the wettability of each film. The hydrophobicity or hydrophilicity of a film’s surface is characterized by the water contact angle. There are no significant differences between the films without BEO; the contact angle of Ch/HNTs films increased with increasing HNT concentration (43.90–58.03°) and, due to the hydrophobic nature of BEO, BEO in nanocomposite films considerably increased (*p* < 0.05) contact angle (52.86–62.85°). Contact angle on the nanocomposite film surface increased comparably after the addition of hydrophobic materials (essential oils) [5,37]. Water contact angle decreases with increasing HNT concentration in Ch/BEO films. On drying, the incorporated HNT molecules in the Ch matrix prevent BEO from agglomerating and floating, resulting in decreased hydrophobicity of the film surface.

### 2.4. Energy Dispersive X-ray Spectroscopy (EDS) Analysis

The elemental composition of the Ch/BEO/HNTs film was analyzed by energy dispersive X-ray spectroscopy (EDS). Figure S2 demonstrates that the HNTs were present in the nanocomposite film. In the Ch/BEO/HNTs film, four distinct peaks were seen. The EDS spectra of the Ch/BEO/HNTs film showed the presence of Si and Al elements. The existence of HNTs on the surface of the Ch film was confirmed by the presence of Al and Si peaks at 1.50 eV and 1.75 eV, respectively [38].

### 2.5. Attenuated Total Reflectance–Fourier Transform Infrared Spectroscopy (ATR–FTIR)

ATR–FTIR spectroscopy was used to evaluate the functional groups between Ch and HNTs in synthesized nanocomposite films. Figure 7c shows the ATR–FTIR spectrum of a Ch/BEO/HNT film with various HNT concentrations. The characteristic absorption bands at 3697 and 3623 cm^−1^ correspond to an HNTs peak O-H bonding stretching vibration, of an interior surface of Al-OH grouping and the O-H bond stretching vibration of the internal Al-OH group between the contact of a Si-O tetrahedron as well as the Al-O octahedron [38,39]. The band at 3282 cm^−1^ is attributed to the O-H stretching vibration. Due to the C-H asymmetric and symmetric stretching vibration, the bands at 2923 and 2879 cm^−1^ appear. The bands at 1645 and 1558 cm^−1^ are attributed to a C=O stretching (amide I) and N-H bending as well as stretching (amide II) [33]. A band at 1409 cm^−1^ may be associated with O-H bending vibration [33]. The band at 1378 cm^−1^ indicates C-N stretching (amide III) [40]. Bands at 1251 and 1153 cm^−1^ are attributed to C-O-C asymmetric stretching [33]. The band at 1029 cm^−1^ is attributed to the Si-O in-plane stretching vibration [38]. The bands at 555 and 460 cm^−1^ are attributed to Al-O-Si and Si-O-Si bonds [38]. There was no change in position in major peaks after the incorporation of HNTs and BEO or their combination. However, there was a slight shift in the peak position, 1558 cm^−1^, of Ch, which moves to 1570 cm^−1^ after mixing HNTs and BEO or their combination; this shows that HNTs and BEO interacted with the amide II bond of Ch.

### 2.6. X-ray Diffraction (XRD)

The crystalline and amorphous domains of Ch/BEO/HNTs nanocomposite films were confirmed using XRD patterns. In this case, it helps to show how HNTs and the biopolymer matrix interact with each other. Figure 7d illustrates the XRD patterns of the synthesized Ch/BEO/HNTs nanocomposite film. The Ch exhibited its distinctive peaks at 2θ = 14.20° and 16.86° [1], and these peaks were generated by the hydrogen bonding of the Ch. The synthesized Ch was found to have partially crystalline and partially amorphous structure, as observed from the peaks. Its crystalline nature was identified during synthesis in Ch/BEO/HNTs, and HNTs exhibit diffraction peaks at 2θ = 20° and 25° [41]. This indicates that the Ch/BEO/HNTs nanocomposite film was successfully formed.

### 2.7. Optical Properties of the Films

The optical barrier in packaging films serves to decrease impact of light on the food components. Figure 7e and Table 1 depict the light transmission and opacity values of the films. The results demonstrated that incorporating BEO and increasing the content of HNTs in the Ch films significantly decreased UV and visible light transmittance across all tested wavelengths. When the Ch/HNTs film was combined with BEO, no transmittance was observed at the UV-C and UV-B wavelengths. The optical path can be obstructed by the presence of HNTs and BEO in Ch films. According to other research, the concentration of nanoparticles in the Ch films resulted in lower light transmission and light diffusion [2,42]. The Ch/BEO/HNTs film allows less than 2% of UV light in the 200–350 nm wavelength range to pass through, indicating that films containing HNTs and BEO have good UV light barrier properties. With the incorporation of BEO, the % transmission of the films was significantly decreased in the visible area 400–800 nm wavelength range. This might be due to light scattering by BEO droplets scattered throughout the polymer films. The results show that the Ch/BEO/HNTs nanocomposite film containing a 30% weight concentration of HNTs has the maximum light barrier properties. The UV light barrier function is significant for preserving food because nutritional loss, lipid oxidation, and discoloration induced by UV radiation may cause packaged foods to decay and lose quality. UV-C light has wavelengths of 100–280 nm, UV-B light has wavelengths of 280–315 nm, and shorter wavelength radiations are almost fully absorbed by the upper and mid atmosphere [5]. Due to its lower sensitivity to ozone in the environment, UV-A light, with a larger spectrum of 315–400 nm, may affect food items. The results suggest the possible use of Ch/BEO/HNTs film in food packaging systems as a UV light barrier film.

In addition, as seen in Figure 7f, increasing the amount of HNTs in the films resulted in an increase in the films’ opacity from 1.51 to 3.34 [43]. In the interior structures, there may have been an interaction between Ch and HNTs that induced light scattering and reflection, which increased the opacity values of the films. Furthermore, the inclusion of BEO had a considerable impact on the film’s opacity. The Ch film seemed translucent and clear. The films containing BEO and HNTs exhibited much higher opacity than that of the Ch/HNTs films [6]. This could be due to the interior structures; there may have been an interaction between Ch, HNTs, and BEO that induced light scattering and reflection, which increased the opacity values of the films.

### 2.8. Mechanical Properties

Table 2 depicts the TS and EB of Ch/BEO/HNTs nanocomposite films. In the Ch and Ch/BEO films, the influence of HNT concentration on the films’ mechanical properties was studied. The film sample which contains 15 wt% HNTs, without BEO, had the maximum TS (17.40 MPa), while the film sample with 30 wt% HNTs had the lowest TS (12.22 MPa). As the quantity of HNTs in the film was raised, the film’s EB was found to decrease (*p* < 0.05). Previous research has shown that Ch films’ TS increases as the quantity of nanoclay particles increases. This may be due to the hydrogen bonding interaction observed between polymer and clay minerals, and possibly strain-induced orientation of a clay particle layer inside the polymer matrix [5,44,45]. The present study demonstrates a comparable result with the content of HNTs at 15 wt%. The TS of the film was found to be drastically reduced (*p* < 0.05) when a HNT content of more than 15% by weight was used. Despite the ultrasonication treatment, certain HNT particles in the film matrix agglomerate, thus causing damage and ruptures inside the film matrix. Table 2 shows the TS and EB of Ch/HNTs films containing BEO. The TS of the films shows an increasing trend until the HNT content reaches 15% by weight. Subsequently, the TS of the films drastically reduced, similar to the Ch film without BEO. Similarly, on increasing the quantity of HNTs in the film, the EB was found to decrease (*p* < 0.05). Furthermore, as mentioned in Table 2, the film with BEO has a lower % Elongation at Break as compared with the film without BEO. The addition of hydrophobic compounds to hydrophilic polymer films lowers moisture content. Because the water also functions as a plasticizer, reduced moisture level inside the film matrix reduces the flexibility of the film [46].

### 2.9. Antioxidant Properties of the Films

**Figure 8:**
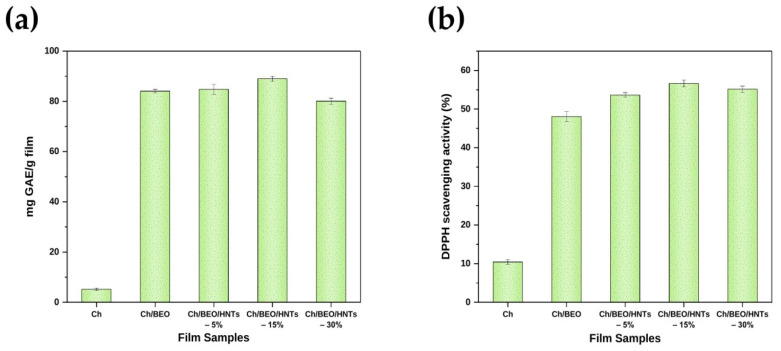
**Antioxidant activity of Ch films formulated with HNTs and BEO.** (**a**) Total phenolic content; (**b**) DPPH scavenging activity.

The total phenolic content of HNTs and BEO formulated Ch films was determined. For Ch/HNTs films containing BEO, the total phenolic contents of the film were evaluated (82.09–88.92 mg GAE/g) (Figure 8a). Because of its phenolic components, the BEO showed the strongest antioxidant activities. Methyl cinnamate, linalool, β-element, and camphor are major constituents of BEO [47]. Many researchers have looked at the influence that the quantity of the active component has on the antioxidant activities of Ch-based films [48]. Furthermore, several studies have reported that the amount of active agent present on the film’s surface is influenced by HNTs. The total phenolic content of the nanocomposite film showed a rise (*p* < 0.05) with the increase in HNT concentration up to 15 wt% (Figure 8a). The inclusion of HNT particles inside the Ch film prevented BEO evaporation from the film surface during drying, because the HNT particles inside the Ch film tend to stabilize the BEO droplets. The total phenolic content of the film was reduced when the HNT content was greater than 15% by weight. This may be due to the uneven distribution of HNT particles and their possible aggregation when concentration increases to more than 15 wt%, as evident from the SEM images given in Figure 6. The HNT particles agglomerate and cause damage and cracks in the film matrix, causing BEO loss from the film [35]. Similar results were obtained with the DPPH assay, as depicted in Figure 8b. The antioxidant properties of the Ch/BEO films containing 15% HNTs by weight were found to be the highest.

### 2.10. Storage Test for Food

**Figure 9:**
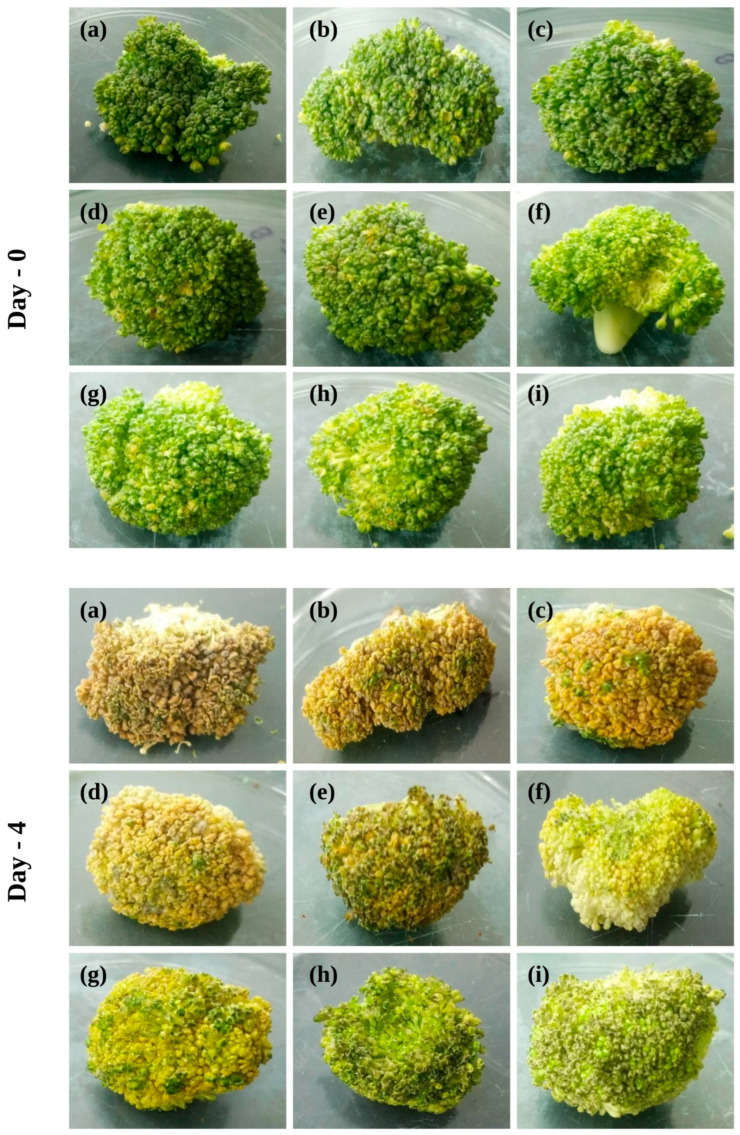
**Broccoli florets after being stored for 4 days.** (**a**) Broccoli florets stored directly; (**b**) Ch film; (**c**) Ch/HNTs—5% (**d**) Ch/HNTs—15%; (**e**) Ch/HNT—30%; (**f**) Ch/BEO; (**g**) Ch/BEO/HNTs—5%; (**h**) Ch/BEO/HNTs—15%; (**i**) Ch/BEO/HNTs—30%.

The results of a study of broccoli’s weight loss during a 4-day storage period are given in Figure 9. Broccoli florets that were stored directly had a significantly greater weight loss than those that were packed in nanocomposite film. The weight loss in broccoli florets that were stored directly was observed to be 63.22%, whereas those kept in Ch/HNTs films showed weight losses ranging from 51.25 to 40.98%, as depicted in Figure 7b. Furthermore, in comparison with films containing BEO and HNTs, broccoli florets kept in neat Ch film lost a substantial amount of weight. When compared with the broccoli florets that were kept in Ch/HNTs films, the broccoli florets that were stored in Ch/BEO/HNTs films went through weight losses ranging from 41.07 to 34.34%.

A comparison of present work with previously reported work is shown in Table 3. These observations suggest that, due to their antioxidant qualities as well as improved barrier capabilities, the Ch/BEO/HNTs films exhibit a great degree of potential for usage in the food sector. This is the first ever report on the creation of a nanocomposite employing a combination of BEO and HNTs for food packaging. In addition, the film’s low-cost creation (Supplementary File) and ecofriendly methodology make it sufficiently acceptable for later commercialization and research.

## 3. Materials and Methods

### 3.1. Materials

Chitosan (Ch) from shrimp shells with deacetylation of 75% was purchased from HiMedia Laboratories Pvt. Ltd. (Mumbai, India). Halloysite nanotubes (HNTs) were purchased from Sigma-Aldrich (Bangalore, India), and basil essential oil (BEO) was purchased from John Aromas Co. (New Delhi, India). Acetic acid, methanol, and glycerol as a plasticizer were purchased from HiMedia Laboratories Pvt. Ltd. (Mumbai, India). Folin & Ciocalteu’s phenol reagent was obtained from Central Drug House (P) Ltd. (New Delhi, India). Sodium chloride, sodium hypochlorite, and DPPH (2,2-Diphenyl-1-picrylhydrazyl) were purchased from HiMedia Laboratories Pvt. Ltd. (Mumbai, India).

### 3.2. Methods

#### 3.2.1. Solution Preparation for Ch/BEO/HNTs

In 100 mL of aqueous acetic acid solution (1% *v*/*v*), Ch powder (1 g) was dissolved overnight at room temperature to generate a 1% *w*/*v* Ch solution. After that, the solution was filtered to eliminate any remaining contaminants. Glycerol was used as a plasticizer at a ratio of 0.40 mL per gram of Ch to allow film formation. Appropriate concentrations of HNTs (0, 5, 15, & 30 wt% relative to Ch powder) were vigorously stirred (REMI 2-MLH, Mumbai, India) in the Ch solution overnight to make nanocomposite dispersions. After that, 500 µL of BEO was progressively added to a 50 mL volume of Ch/HNTs mixture while gently stirring. In an ice bath (3 ± 1 °C), dispersions of Ch/HNTs with and without BEO were homogenized for 10 min with 30 percent vibration amplitude using an ultrasonic homogenizer (Ultrasonic cleaner, 230-HTD, Shenzhen, China). To eliminate bubbles, the film-forming solutions were vacuum degassed for 15 min.

#### 3.2.2. Fabrication of Nanocomposite Films

The 50 mL Ch/HNTs mixtures, either with or without BEO, were poured and casted into 90 mm Petri plates. The samples were dried for 96 h at ambient temperatures (25 °C with 50% relative humidity). After 96 h, the casted films were peeled off and stored at room temperature.

### 3.3. Characterization of Films

#### 3.3.1. Thickness of the Films

An electronic micrometer instrument (Digimatic Micrometer 293-821, Mitutoyo Corporation, Tokyo, Japan) with a 0.01-mm precision was used to determine the thickness of each film composition. On each film, measurements were taken at three distinct points.

#### 3.3.2. Moisture Content

The moisture content of the film samples (2 cm × 2 cm) was calculated by letting the film samples dry at 110 °C until the weights were uniform and then observing the difference.

#### 3.3.3. Water Absorption and Solubility

The water absorption and solubility of the films were measured gravimetrically [35]. Every film sample with a dimension of 2 cm^2^ was dried in a hot air oven at 110 °C until they reached uniform weight. After that, each dried film sample was soaked in 25 mL of deionized water for 24 h at 25 °C in a water bath. Then the film samples were removed and wiped with the help of tissue paper to remove surface water, and weighed thereafter. The remaining film samples were further dried at 110 °C until consistent weight was achieved.

The following formula was used to determine the water absorption and solubility of the films:

(1) $$\mathrm{Water}\;\mathrm{absorption}\;(\%)=\frac{A_{2}-A_{1}}{A_{1}}\times 100$$

(2) $$\mathrm{Solubility}\;(\%)=\frac{A_{1}-A_{3}}{A_{1}}\times 100$$

The weight values of the film before and after it was immersed were A_1_ and A_2_, respectively. A_3_ was the final dried film sample’s weight.

#### 3.3.4. Contact Angle

A contact angle goniometer instrument (OCA 20 Physics Instrument, Filderstadt, Germany) was used to examine the advancing water contact angle. For this purpose, a 10 µL deionized water droplet was placed on the surface of the film to evaluate the film’s surface hydrophobicity. The film samples were conditioned for three days before the measurement. The measurement was performed at 25 °C with a relative humidity of 50%, and the rectangular sized film (5 × 1 cm) was mounted on the top of a solid plane support and kept stable during the examination. The measurements were taken within the first 10 s after dropping deionized water on the surface of the film.

### 3.4. Scanning Electron Microscopy (SEM)

The dispersion of oil pores and HNTs in the film samples was examined using Scanning Electron Microscopy (MIRA3 TESCAN, Harrisburg, PA, USA) to investigate the surface morphology of the film samples. After mounting the films on aluminum stubs, gold coating was applied to the mounted samples.

### 3.5. Energy Dispersive X-ray Spectroscopy (EDS)

The elemental composition of the Ch/BEO/HNTs film was determined by using energy dispersive X-ray spectroscopy (X-Flash Detector 5010, Bruker, Nano GmbH, Berlin, Germany) equipped with SEM.

### 3.6. Attenuated Total Reflectance–Fourier Transform Infrared Spectroscopy (ATR–FTIR)

The ATR–FTIR spectra of the Ch, Ch/HNTs, and Ch/HNTs/BEO films were measured using an ATR–FTIR spectrophotometer (60 MHz Varian 360, Perkin Elmer, Waltham, Mass, USA). Each spectrum was measured between 400 and 4000 cm^−1^.

### 3.7. X-ray Diffraction (XRD)

The XRD pattern analyses of Ch, Ch/HNTs, and Ch/HNTs/BEO films were performed using an X-ray Diffractometer (DMax 2200, Rigaku, Tokyo, Japan) to identify their structure. The film samples were tested at a rate of 3°/min ranging from 2 theta = 0 to 80°.

### 3.8. Optical Properties of the Film

The percentage of UV light and visible light transmission through the polymer matrix was determined using a UV-Vis spectrophotometer (HACH DR6000, Aurora, CO, USA). The light transmission of film samples was measured between 200–800 nm. A rectangular-shaped piece of film was inserted into a cuvette for analysis. Based on the film’s absorbance at 600 nm, the following equation was used to calculate the film’s opacity [2]:

y = A/x (3)

where A was the absorbance of the film at 600 nm, x was the thickness of the film measured in mm, and y (mm^−1^) represents the film’s opacity.

### 3.9. Mechanical Properties

The nanocomposite films’ tensile strength (TS) and % Elongation at Break (EB) were evaluated using Type Specimens in the 5KN Capacity (Shimadzu UTM AGS-J, Singapore) as per the ASTM Standard Method D 638 [50]. Each specimen was tested with the crosshead speed of 5 mm/min and the initial grip separation of 50 mm. For each film sample, three specimens were evaluated.

### 3.10. Antioxidant Properties

#### 3.10.1. Total Phenolic Content

To evaluate the total phenolic content of the Ch/BEO/HNTs film, the Folin–Ciocalteu method was performed [51]. In 5 mL of methanol, 80 mg of the film was dissolved, then 20 µL film extract was diluted in 1.58 mL distilled water, after which 0.1 mL Folin–Ciocalteu solution was added and stirred well. A sodium carbonate solution of 300 µL was added after 8 min and mixed well. After that, the solution was kept for 2 h at room temperature in the dark. Then, a UV-Vis spectrophotometer (HACH DR6000, Aurora, CO, USA) was used to measure absorption at 765 nm. The total phenolic content was measured in Gallic acid equivalents mg GAE/g of film.

#### 3.10.2. DPPH (2,2-Diphenyl-1-picrylhydrazyl) Assay

The DPPH free radical scavenging test was used to determine the antioxidant properties of the film samples. The measurements were performed in accordance with the technique defined by Siripatrawan et al. [48] with slight modification. In 5 mL of methanol, 80 mg of the film was dissolved, and then 400 µL film extract was retrieved and diluted in 1.60 mL of DPPH methanolic solution. Then, the solution was kept for 30 min at 25 °C in a dark place. Then, a UV-Vis spectrophotometer (HACH DR6000, Aurora, CO, USA) was used to measure absorption at 517 nm. The % of DPPH free radical scavenging was calculated using the following formula:

(4) $$\mathrm{DPPH}\;\mathrm{scavenging}\;\mathrm{activity}\;(\%)=\frac{A_{DPPH}-A_{extract}}{A_{extract}}\times 100$$

The absorbance value of DPPH methanolic solution was A_DPPH_, whereas the absorbance value of film extract was A_extract_.

### 3.11. Storage Test for Food

The broccoli was purchased fresh from the market. The broccoli was of consistent size and color, free of physical damage and fungal contamination. Florets were cleaned with 1% (*v*/*v*) sodium hypochlorite for 1 min and then washed in distilled water and allowed to air dry for 1 h in the ambient condition [52]. 5 g broccoli florets were also packed in Ch/BEO/HNTs films. For 4 days, samples were kept at 25 °C with 55% relative humidity. Using an equation, the weight loss of broccoli florets was calculated. The weight decrease was expressed as a percentage (%).

(5) $$\mathrm{Weight}\;\mathrm{loss}\;(\%)=\frac{W_{i}-W_{f}}{W_{i}}\times 100$$

where *W_i_* represents the weight on the day of packaging and *W_f_* represents the weight after unpacking on the testing day.

### 3.12. Statistical Analysis

Statistical analysis of experimental data was carried out using Origin software (OriginLab Corporation, Northampton, MA, USA) to estimate a one-way ANOVA. All the tests were carried out in triplicates (*n* = 3), and the data are shown as mean ± standard deviation. Significant value was *p* < 0.05.

## 4. Conclusions

**Figure 10:**
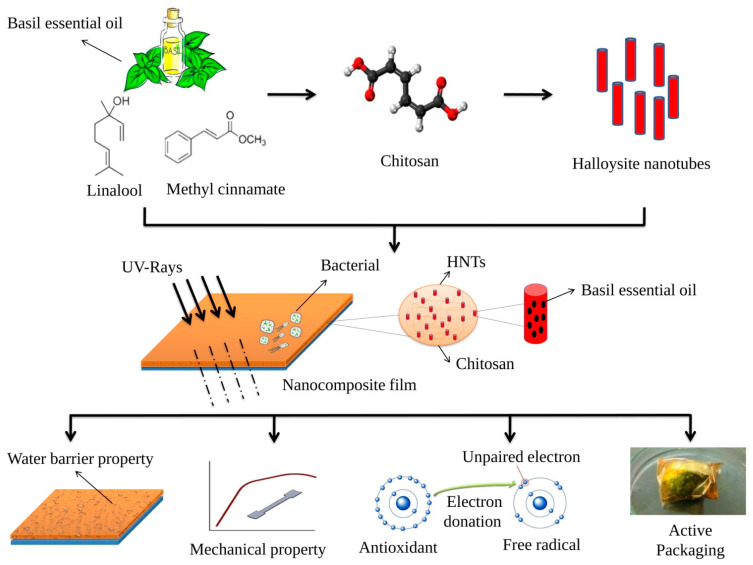
Schematic representation showing mechanism of Ch films formulated with HNTs and BEO improving qualities of the nanocomposite film.

The present study addresses the development of nanocomposite film intended for food packaging applications (Figure 10). The incorporation of BEO and HNTs in a chitosan base enhanced various characteristics of the resultant nanocomposite that make it a potential candidate for food packaging material. The Ch/BEO/HNTs nanocomposite with higher HNT concentration has significant barrier properties and antioxidant properties that delayed the decay of the test food material. Given the substantial results obtained with this Ch/BEO/HNTs nanocomposite, there is a need for further research to develop the nanocomposite for multipurpose use and to make the technology more cost-effective.

## Figures and Tables

**Table 1:** Light transmission values of Ch film synthesized with the HNTs and BEO were determined at 200 nm (UV-C), 300 nm (UV-B), 400 nm (UV-A), and 600 nm (Visible).

Sample	wt% HNTs	%Transmission at (200 nm UV-C)	%Transmission at (300 nm UV-B)	%Transmission at (400 nm UV-A)	%Transmission at (600 nm Visible)
**Ch**	0	0	3.20	42.10	73.70
**Ch/HNTs**	5	0	0.40	14.30	33.70
	15	0	0.20	7.20	19.20
	30	0	0	3.90	13.40
**Ch/BEO**	0	0	0	3.40	46.00
**Ch/BEO/HNTs**	5	0	0	15.20	58.00
	15	0	0	5.20	27.10
	30	0	0	1.50	10.70

**Table 2:** Tensile strength and elongation at break of the films.

Samples	wt% HNTs	Tensile Strength (MPa)	% Elongation at Break
**Ch**	0	11.37 ± 1.41	2.02 ± 0.50
**Ch/HNTs**	5	13.51 ± 0.83	1.56 ± 0.43
	15	18.52 ± 1.04	1.06 ± 0.38
	30	12.81 ± 0.66	1.02 ± 0.58
**Ch/BEO**	0	8.56 ± 1.12	1.84 ± 0.43
**Ch/BEO/HNTs**	5	10.28 ± 0.94	0.85 ± 0.33
	15	13.31 ± 0.74	0.62 ± 0.15
	30	9.22 ± 1.04	0.52 ± 0.22

**Table 3:** Comparative study of active food packaging material with previously reported work.

Polymer	Nanofiller	Active Agent	Method	UV Light %Trans-mission at (600 nm Visible)	Opacity (mm^−1^)	Moisture Content (%)	References
Chitosan	Halloysite Nanotubes	Basil oil	Solution casting	10.17	5.20	17.51	Present Study
Chitosan	Zinc oxide nanoparticles	Neem oil	Solution Casting	NR	3.69	NR	[1]
Chitosan	Zinc oxide	Artemisia annua oil	Solution Casting	NR	1.34	8.77	[49]
Chitosan	Silver nanoparticles	Citrus extract	Solution Casting	17.40	NR	NR	[33]

## Data Availability

The authors confirm that the data supporting the findings of this study are available within the article.

## Supplementary Materials

The following supporting information can be downloaded at: https://www.mdpi.com/article/10.3390/antibiotics11121820/s1. Figures S1 and S2: SEM and EDX analysis of the films and cost estimation.

## Abbreviations

BEO, Basil essential oil; HNTs, Halloysite nanotubes; Ch, Chitosan; SEM, Scanning electron microscopy; XRD, X-ray diffraction; FTIR, Fourier transform infrared; DPPH, 2,2-diphenyl-1-picrylhydrazyl; GRAS, Generally recognized as safe.
