# Supplementary material for: Fabrication and Evaluation of Basil Essential Oil-Loaded Halloysite Nanotubes in Chitosan Nanocomposite Film and Its Application in Food Packaging
Source: Antibiotics (Basel). 2022 Dec 15;11(12):1820. doi: 10.3390/antibiotics11121820 (PMC9774598; doi:10.3390/antibiotics11121820)
Supplement: Supplementary file 1 [file antibiotics-11-01820-s001.zip › Supplementary File.pdf]

Fabrication and Evaluation of Basil-Essential Oil Loaded Halloysite-Nanotubes in Chitosan Nanocomposite Film and Its Application in Food Packaging

Narayan Chaudhary [1], Gourav Mishra [1,*], Tushar Yadav [2], Nishant Srivastava [1], Vimal K. Maurya [3] and Shailendra K. Saxena [3,*]

[1] Nanotoxicity & Drosophila Research Laboratory, Department of Biotechnology, Meerut Institute of Engineering and Technology, Meerut 250005, India.
[2] Department of Zoology, Jawaharlal Nehru Smriti Government Postgraduate College, Shujalpur 465333, India.
[3] Centre for Advanced Research (CFAR), Faculty of Medicine, King George's Medical University (KGMU), Lucknow 226003, India.http://orcid.org/0000-0003-2856-4185
* Corresponding Authors: *mishragourav88@gmail.com (GM) https://orcid.org/0000-0001-8060-5277; *shailen@kgmcindia.edu; Tel: 91 522 2257450; Fax: 91 522 2257450; http://orcid.org/0000-0003-2856-4185 (SKS)

1. Scanning electron microscope (SEM)

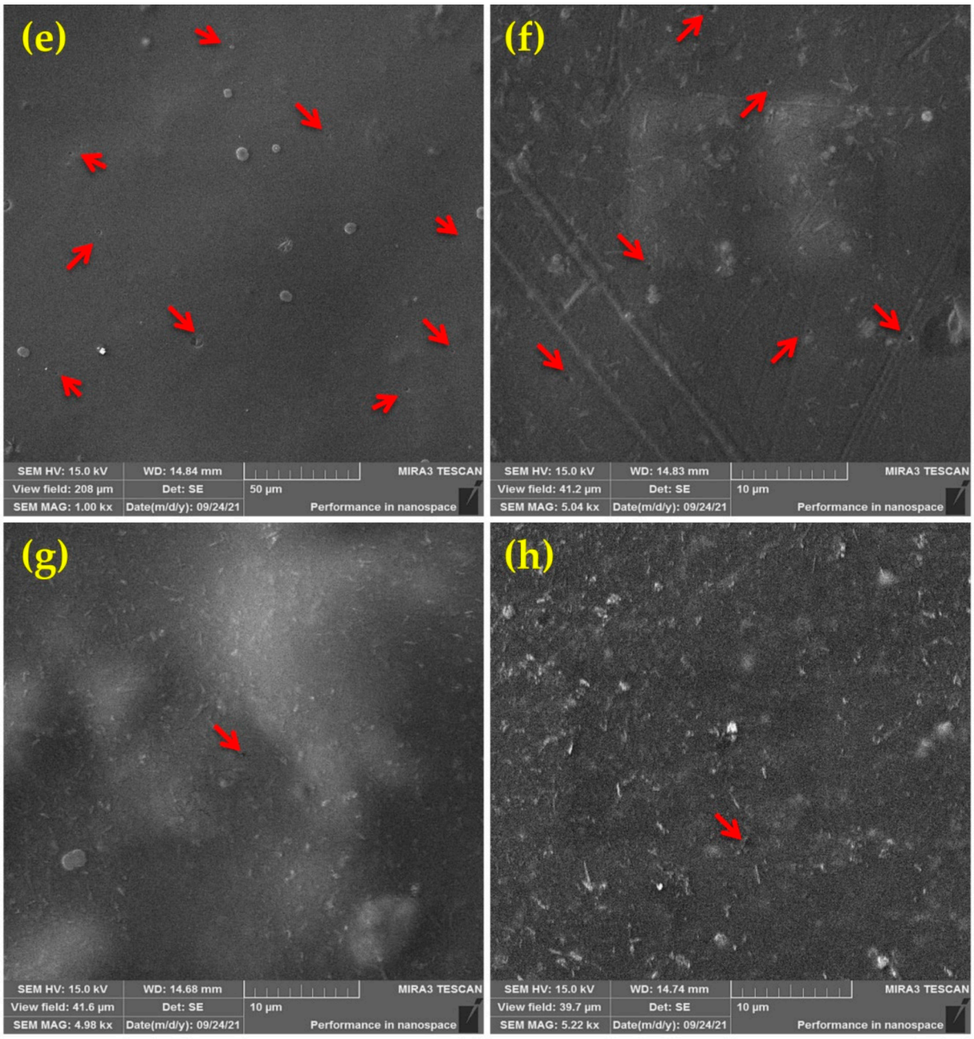

Figure S1. Scanning Electron Microscopy (SEM) images of the films (e) Ch/BEO (f) Ch/BEO/HNTs - 5 % (g) Ch/BEO/HNTs - 15 % (h) Ch/BEO/HNTs - 30 %.

2. Energy Dispersive X-ray Spectroscopy (EDS) Analysis

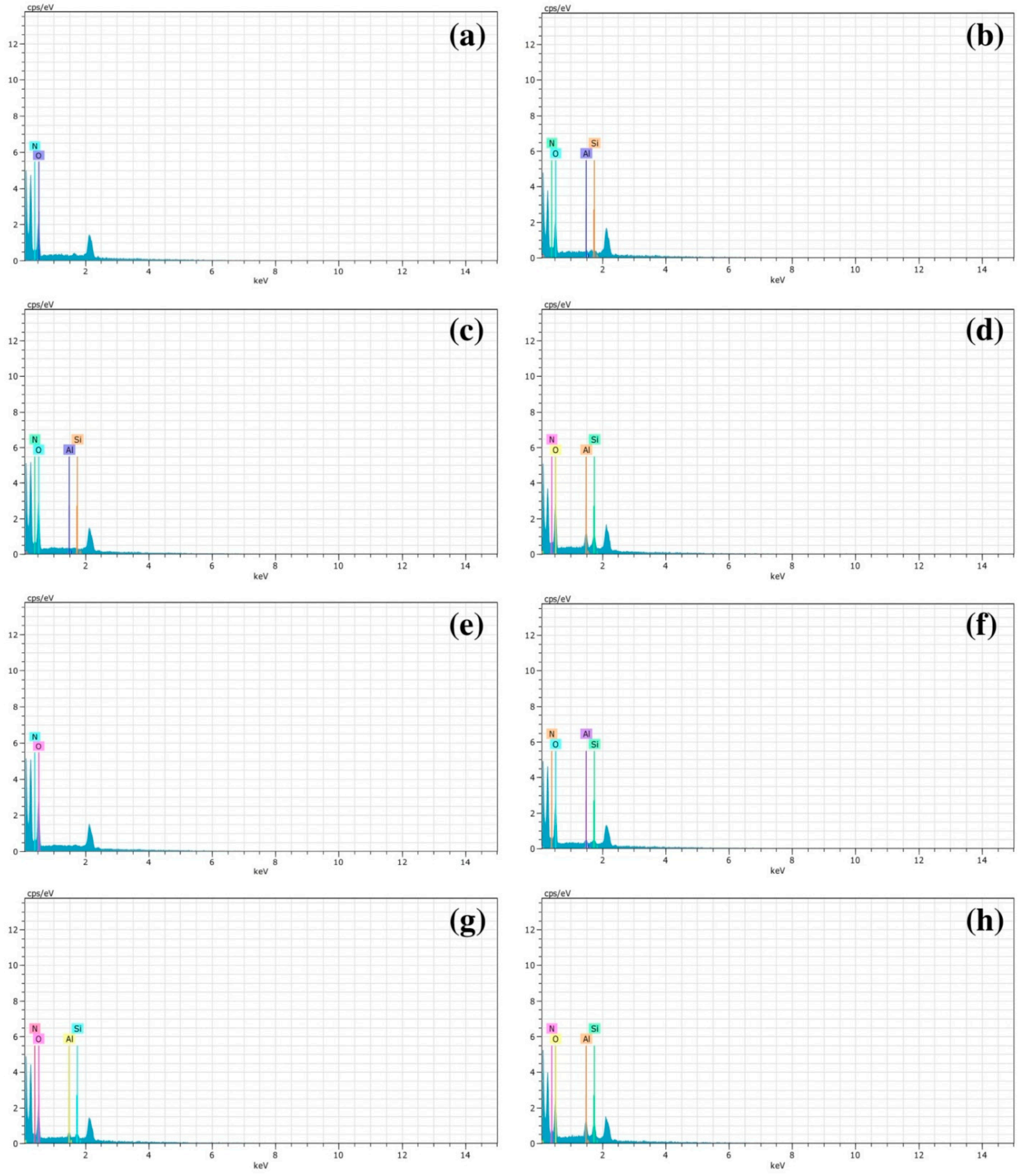

Figure S2. Energy Dispersive X-ray Spectroscopy (EDS) analysis of the films (a) Ch film (b) Ch/HNTs - 5 % (c) Ch/HNTs - 15% (d) Ch/HNTs - 30 % (e) Ch/BEO (f) Ch/BEO/HNTs - 5 % (g) Ch/BEO/HNTs - 15 % (h) Ch/BEO/HNTs - 30 %.

3. Cost estimation.

Cost of 9 cm^2 film

- Cost of Chitosan (cost of 100 g chitosan polymer (HiMedia) is Rs 3422, therefore cost of 0.5 g of chitosan polymer INR 17)
- Cost of Halloysite Nanotubes (HNTs) wt15% (cost of 100 g of HNTs (Sigma-Aldrich) is INR 3500, therefore cost of 0.15 g INR 5)
- Cost of Basil Essential Oil (BEO) (cost of 250 ml of BEO (John Aromas Co.) is INR 462, therefore cost of 0.5 ml is approx INR 1)
- Cost of Acetic acid (cost of 500 ml of acetic acid is INR 366 (HiMedia), therefore cost of 0.5 ml is approx INR 1)
- Cost of Glycerol (cost of 500 ml of glycerol is INR 535 (HiMedia), therefore cost of 0.2 ml is approx INR 1)

Utilities

- Magnetic Stirrer 500 watt
- DI water 50 ml = INR 1

The cost of magnetic stirrer used for 12 h = power kW x time (h) x cost of electricity

= 0.550 x 12 x 5

= 33 INR

Therefore, the cost of polymer film

**Materials with quantity**	**Cost (INR)**
0.5 g chitosan	17
0.15 g HNTs	5
0.5 ml BEO	1
0.5 ml acetic acid	1
0.2 ml glycerol	1
50 ml DI water	1
Electricity	33
**Total**	**59 INR**
